# P-1048. An Intervention to Remove or De-Escalate to the Lowest Infection Risk Catheters: Impact on Device Utilization

**DOI:** 10.1093/ofid/ofaf695.1243

**Published:** 2026-01-11

**Authors:** Shruti K Gohil, Jennifer Yim, Keith M Madey, Kathleen A Quan, Jordan Oliver, Maurice Espinoza, Lisa Wilhelm, Amarah Mauricio, Thomas T Tjoa, Allen Kong, Jennifer Cox, Joe Carmichael, Susan Huang

**Affiliations:** University of California, Irvine, Irvine, CA; Epidemiology & Infection Prevention, UC Irvine Health, Orange, CA, Orange, California; University of California, Irvine, Irvine, CA; University of California, Irvine, Irvine, CA; UC Irvine Medical Center, UCI Health, Orange, CA, Orange, California; Nursing Education, UC Irvine Health, Orange, CA, Orange, California; Nursing Education, UC Irvine Health, Orange, CA, Orange, California; Division of Infectious Diseases, University of California, Irvine School of Medicine, Irvine, California; University of California, Irvine School of Medicine, Division of Infectious Diseases, Irvine, California; Nursing Education, UC Irvine Health, Orange, CA; UC Irvine School of Medicine, Department of Surgery, Orange, California; University of California Irvine, School of Medicine, Irvine, CA, Division of Infectious Diseases, Irvine, California; Nursing Education, UC Irvine Health, Orange, CA; UC Irvine School of Medicine, Department of Surgery, Orange, California; University of California, Irvine School of Medicine, Irvine, California

## Abstract

**Background:**

Most hospitalized patients do not need central venous catheters (CVCs) and can be safely managed with catheters that have lower central line associated bloodstream infection (CLABSI) risk, such as peripherally inserted central catheters (PICCs), midlines, and peripheral intravenous (PIV) catheters. We evaluated the impact of a catheter selection and de-escalation program that included electronic nursing and physician assessment of catheter necessity on device use.

**Figure 1:**
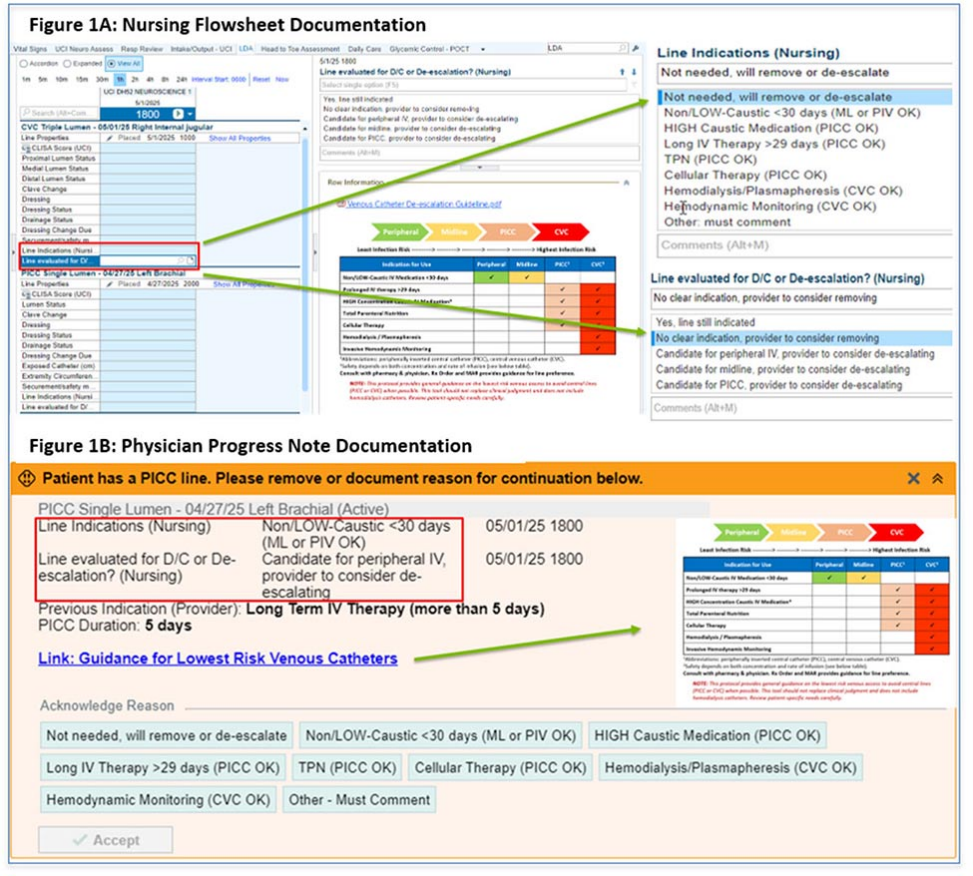
Nursing Electronic Medical Record Figure 1: Flowsheet Assessment of Catheters Eligible for De-Escalation with Automated Physician Progress Note Flagging and Documentation of Line Necessity Figure 1: (A) Nursed enter daily assessment of line indication and evaluate for removal or de-escalation to a line with lower infection risk. (B) Physician progress note auto-populates with notification from nurse flagging potential candidates for catheter removal/de-escalation.

**Figure 2:**
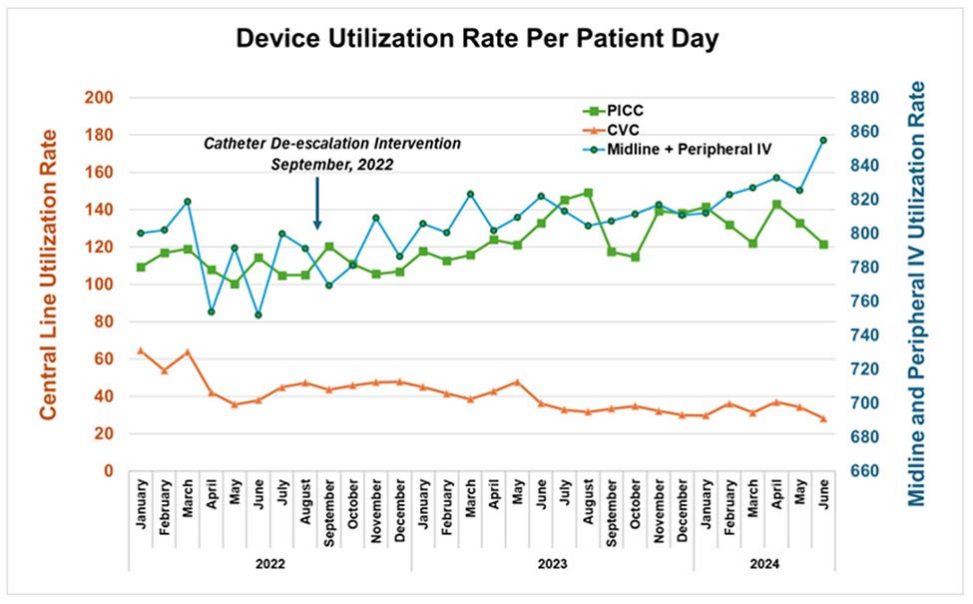
Device Utilization Rate Per Patient Day Before and After De-Escalation Intervention Figure 2: Monthly device utilization rates (catheter-days divided by patient-days ) before and after implementation of a catheter de-escalation program consisting of (a) electronic nursing assessments of central venous catheters that could be removed or de-escalated to a catheter with lower infection risk (PICC, midline, or peripheral IV), (b) automated notification within physician progress notes for documentation of line necessity, and (c) education.

**Methods:**

In this prospective cohort study, we evaluated hospitalized adults with CVCs, PICCs, midlines, and PIVs at a large academic medical center during baseline (1/2022-8/2022) and after intervention (9/2022-6/2024), consisting of: (1) daily nursing assessment and electronic health record documentation of catheters for removal or de-escalation (e.g., from CVC to PICC, or from PICC to midlines/PIVs), (2) automated cascading of potential candidates for de-escalation into physician progress notes, and (3) nursing/physician education to use catheters with the lowest infection risk (Figure 1). We assessed demographics, device type, and device utilization rates (DUR, catheter-days/patient-days). T-test compared mean baseline and intervention DURs.

**Results:**

Across the study period there were 46,542 patients (692,649 patient-days), mean (SD) age was 59.4 (17.2), 58% (26761) were male. There were 4,074 CVCs, 4,133 PICCs, 1,985 midlines, and 48,318 PIVs. CVC DURs decreased 23% (p< 0.01) from mean (SD) 48.8 (10.3) to 37.7 (6.3) during the baseline and intervention periods, respectively. PICC DURs increased 15% (p< 0.01) from 108.7 (6.1) to 125.7 (12.7); midline DURs increased 9% from 41.6 to 45.3 (p=0.10); PIV DURs increased 2.6% (p=0.05) from 788.7 to 811.3 (Figure 2).

**Conclusion:**

A central line de-escalation protocol that included electronic flagging of CVCs that could be de-escalated to less invasive catheters with lower infection risk, coupled with education, successfully reduced CVC utilization.

**Disclosures:**

Susan Huang, MD, MPH, Xttrium: Conducting studies in which participating nursing homes and hospitalized patients receive contributed antiseptic products|Xttrium Laboratories: Conducting studies in which participating nursing homes and hospitalized patients receive contributed antiseptic product

